# Exoskeleton gait training on real-world terrain improves spatiotemporal performance in cerebral palsy

**DOI:** 10.3389/fbioe.2024.1503050

**Published:** 2024-12-17

**Authors:** Emmanuella A. Tagoe, Ying Fang, Jack R. Williams, Julie L. Stone, Zachary F. Lerner

**Affiliations:** ^1^ Department of Mechanical Engineering, Northern Arizona University, Flagstaff, AZ, United States; ^2^ Department of Physical Therapy and Athletic Training, Northern Arizona University, Flagstaff, AZ, United States; ^3^ College of Medicine – Phoenix, University of Arizona, Phoenix, AZ, United States

**Keywords:** cerebral palsy, exoskeleton, gait training, low-frequency, spatiotemporal, real-world

## Abstract

**Introduction:**

Walking is essential for daily life but poses a significant challenge for many individuals with neurological conditions like cerebral palsy (CP), which is the leading cause of childhood walking disability. Although lower-limb exoskeletons show promise in improving walking ability in laboratory and controlled overground settings, it remains unknown whether these benefits translate to real-world environments, where they could have the greatest impact.

**Methods:**

This feasibility study evaluated whether an untethered ankle exoskeleton with an adaptable controller can improve spatiotemporal outcomes in eight individuals with CP after low-frequency exoskeleton-assisted gait training on real-world terrain.

**Results:**

Comparing post- and pre-assessment, assisted walking speed increased by 11% and cadence by 7% (*p* = 0.003; *p* = 0.006), while unassisted walking speed increased by 8% and cadence by 5% (*p* = 0.009; *p* = 0.012). In the post-assessment, assisted walking speed increased by 9% and stride length by 8% relative to unassisted walking (*p* < 0.001; *p* < 0.001). Improvements in walking speed were more strongly associated with longer strides than higher cadence (*R* ^2^ = 0.92; *R* ^2^ = 0.68). Muscle activity outcomes, including co-contraction of the soleus and tibialis anterior, did not significantly change after training.

**Discussion:**

These findings highlight the spatiotemporal benefits of an adaptive ankle exoskeleton for individuals with CP in real-world settings after short-term training. This work paves the way for future randomized controlled trials (RCTs) to evaluate the isolated effects of adaptive ankle exoskeletons on gait performance and neuromuscular outcomes in individuals with CP in real-world environments

## 1 Introduction

Walking is often challenging for individuals with neurological conditions like cerebral palsy (CP). CP remains the most widespread childhood walking disability globally (Michael-Asalu et al., 2019; Graham et al., 2016). Ambulatory people with CP walk at less than half of the typical speed of their peers, with similar reductions in stride length and cadence (Kim and Son, 2014). This limits their social activity and participation levels (Warren et al., 2016), posing a significant challenge to their quality of life. Moreover, unlike their typically developing peers, individuals with CP have a reduced ability to increase stride length and instead rely on increased cadence as a compensatory strategy to increase walking speed (Abel and Damiano, 1996; Davids et al., 2019). Hence, increasing the walking speed and stride length is the important clinical goal for individuals with CP and their families (Todd et al., 1989; Hoffman et al., 2018).

Furthermore, individuals with CP exhibit significantly higher co-contraction of agonist–antagonist muscles compared to their typically developing peers (Poon and Hui-Chan, 2009; Damiano et al., 2000; van Roon et al., 2005). Although co-contraction of the plantar- and dorsiflexor muscles can be helpful for ankle stability and balance while walking (Di Nardo et al., 2015), elevated co-contraction of these muscles is considered a major cause of inefficient walking in individuals with CP (Winter 1978; Winter 2009). Thus, there is a need for interventions that can reduce the excessive co-contraction of agonist–antagonist muscles in individuals with CP to improve their walking outcomes.

In recent years, exoskeletons have proven to be beneficial for individuals with neurological impairments, particularly while walking on a treadmill or in highly controlled overground environments. These devices typically assist or resist multiple or individual lower-limb joints while walking to compensate for impaired mobility or promote the usage of affected joints (Buesing et al., 2015; Orekhov et al., 2020; Fang et al., 2022; Srivastava et al., 2015; Lerner et al., 2017; Conner B. C. et al., 2021). With recent advancements, they are also capable of adapting to an individual’s specific mobility impairments and provide assistance suitable for different terrain types, improving overall mobility (Slade et al., 2022; Bishe et al., 2021; Gasparri et al., 2019). For instance, the CP Walker, an in-clinic exoskeleton system described by Bayón et al., provides assistance to all lower-limb joints during gait training and has been shown to improve spatiotemporal performance and strength in individuals with CP (Bayón et al., 2018). Similarly, Nakagawa et al. demonstrated that the hybrid assistive limb (HAL), which assists the hip and knee, improves the walking speed and step length in children with CP (Nakagawa et al., 2020). Our previous research also demonstrated that ankle exoskeleton assistance improves gait speed, walking distance, stride length, and muscle activity recruitment (Conner B. et al., 2021; Fang and Lerner, 2022) while reducing ankle co-contraction in individuals with CP after ankle exoskeleton resistance training (Conner B. C. et al., 2021).

Overall, evidence suggests that exoskeleton gait training in controlled environments can lead to increased walking speed and stride length and reduced co-contraction of plantar- and dorsiflexor muscles for individuals with neurological conditions (Fang et al., 2022; Srivastava et al., 2015; Lerner et al., 2017; Conner B. C. et al., 2021; Bayón et al., 2018; Nakagawa et al., 2020; Conner B. et al., 2021; Fang and Lerner, 2022; Awad et al., 2017). However, despite these promising findings, no study to date has demonstrated whether these improvements in spatiotemporal performance and neuromuscular control observed in controlled laboratory settings effectively translate to real-world environments in any impaired population.

The purpose of this feasibility study was to investigate whether short-term, low-frequency training with adaptive ankle exoskeleton plantarflexion assistance on real-world terrain would improve assisted and unassisted spatiotemporal and neuromuscular outcomes in individuals with CP. We hypothesized that walking speed and stride length would increase after exoskeleton-assisted training and would be further enhanced when walking with assistance compared to without assistance. We also hypothesized that exoskeleton-assisted training would reduce co-contraction between agonist and antagonist muscles. To test these hypotheses, eight individuals with CP completed walking assessments before and after two ankle exoskeleton-assisted training sessions on the real-world terrain.

## 2 Materials and methods

### 2.1 Exoskeleton design and control

**FIGURE 1:**
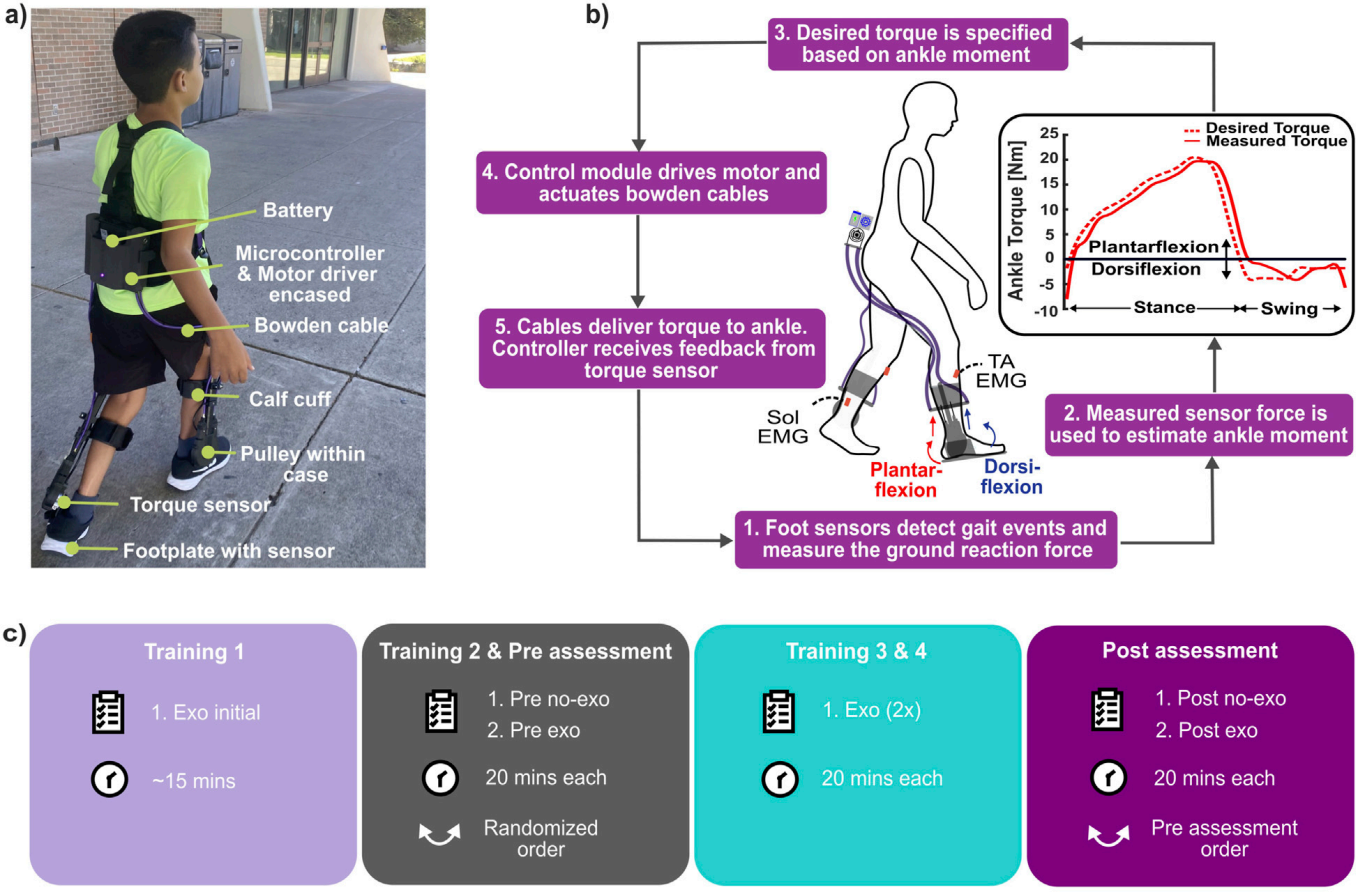
**(A)** Participant wearing our ankle exoskeleton with its components labeled. **(B)** Exoskeleton control and operation. **(C)** Experimental protocol used in the study (Training 1 was untimed exoskeleton-assisted lap estimated to take ∼15 min).

We used an untethered, lightweight, battery-powered ankle exoskeleton (Biomotum, Inc.), which is described in detail by Orekhov et al. (2021). In brief, the device consisted of a motor and battery assembly, Bowden cables, and ankle assemblies that included a footplate embedded with a force sensor, torque sensor, pulley, and calf cuff (Figure 1A). The motor assembly, mounted on the user’s waist, delivered assistive torque to the ankle assembly through compliant Bowden cables, positioning most of the device’s mass proximally in order to avoid the metabolic penalty associated with distally placed mass (Browning et al., 2007).

The exoskeleton utilized a proportional joint-moment control strategy to provide assistance that adapts to specific ankle joint impairments and various terrain types. The controller, therefore, delivered assistive torque proportional to the real-time estimate of the biological ankle joint moment (Equation 1; Figure 1B); details and validation of the control strategy were previously reported by Bishe et al. (2021) and Gasparri et al. (2019). In brief, force sensors on footplates measured plantar pressure, which was then used to estimate the biological ankle moment. This estimation employed a regression equation and a calibration process performed during the first five steps of each walking trial, as shown in Equation 2. The foot sensors were also used to inform a finite state machine that distinguished between the stance and swing phases, enabling the exoskeleton to adapt the participant’s gait pattern, walking speed, and terrain.

$$T_{\text{assistive}}\left(t\right)=t_{\text{setpoint}}M_{\text{ankle}}\left(t\right),$$ (1)

$${M\;}_{\text{ankle}}\;\left(t\right)=1.29\;{R_{F}}^{2}-0.51\;R_{F}+0.22,$$ (2)

where $T_{\text{assistive}}\left(t\right)$ was the exoskeleton assistive torque specified by the controller, $t_{\text{setpoint}}$ was the nominal peak torque setpoint, ${M\;}_{\text{ankle}}\;\left(t\right)$ was the real-time estimate of the biological ankle moment, and $R_{F}$ was the real-time force sensor force ratio calculated as $\left(R_{F}=F_{\text{foot}}/F_{\text{cal}}\right)$ , where $F_{\text{foot}}$ was the instantaneous force sensor force and $F_{\text{cal}}$ was the average peak force sensor force during the calibration process (Bishe et al., 2021; Gasparri et al., 2019).

### 2.2 Participants

Eight individuals with CP, aged between 12 and 27 years old and classified as Gross Motor Function Classification System (GMFCS) level I–III, participated in this study (Table 1). The inclusion criteria for this study were a diagnosis of CP; GMFCS level I, II, or III; body mass up to 60 kg; and the ability to walk overground for at least 20 min with or without a walking aid. Participants were excluded if they had lower-limb orthopedic surgery within the last 6 months, stance phase knee hyperextension during normal or exoskeleton-assisted walking, or any health condition that would prevent the safe completion of this study. A licensed physical therapist performed a physical exam on each participant to assess function and confirm eligibility. A limit for body mass was implemented to allow for a peak torque setpoint of 0.35 Nm/kg (within the operating specifications of the exoskeleton device of a maximum of 21 Nm).

**TABLE 1:** Participant characteristics.

Participant	Age (years)	Sex	Mass (kg)	Height (m)	GMFCS level	More affected side	Gait type
P1	13	M	44.3	1.58	II	Right	Moderate ankle PF dysfunction and unilateral crouch
P2	13	M	57.0	1.67	I	Right	Mild ankle PF dysfunction and bilateral crouch
P3	12	M	32.7	1.42	II	Right	Moderate ankle PF dysfunction and asymmetric crouch gait*
P4	16	M	58.2	1.67	II	Left	Moderate ankle PF dysfunction and bilateral crouch
P5	16	M	55.4	1.74	I	Left	Mild ankle PF dysfunction and bilateral crouch
P6^†^	27	F	42.8	1.47	III	Left	Severe ankle PF dysfunction and bilateral crouch
P7	14	M	51.9	1.61	II	Right	Moderate ankle PF dysfunction and bilateral crouch
P8	12	M	44.3	1.61	I	Right	Mild ankle PF dysfunction and bilateral crouch

GMFCS, Gross Motor Function Classification System; PF, plantarflexion. *P3 has severe crouch gait on the right side and moderate crouch on the left side. ^†^P6 required the use of walker to complete study protocol and, as such, used ramps (inclines) in place of stairs, as expected for GMFCS level III individuals.

### 2.3 Experimental protocol

The study protocol was approved by the Northern Arizona University Institutional Review Board (#986744). All adult participants and parents of participants under 18 provided written consent; minor participants provided verbal assent.

This study utilized a 409-m outdoor walking path with multiple terrains that consisted of level ground, stairs, and approximately 5 ° inclines and declines. The path included 168.5 m of level ground, 74.2 m of inclines, 153.2 m of declines, and two sets of stairs totaling 13.1 m (21 steps). These terrains alternated with five turns that lead back to the starting point. Over a 14-day span, participants completed four walking sessions on the path, including pre- and post-assessments (Figure 1C). In the first session, participants completed an untimed lap with exoskeleton assistance to familiarize themselves with the path and prevent any naivety to the path from artificially biasing baseline measurements. Following this, participants completed the pre-assessment, which involved two 20-minute walks, one with and one without exoskeleton assistance. The testing order was block-randomized across participants. In the next two sessions, participants completed two 20-minute walks with the exoskeleton. In the final session, i.e., the post-assessment, participants again completed two 20-minute walks, one with and another without exoskeleton assistance, following the same order as in the pre-assessment.

Electromyography (EMG) data from the soleus and tibialis anterior (TA) were collected bilaterally at 1259 Hz using a wireless surface electrode system (Trigno, Delsys, Natick, MA) during pre- and post-assessments. A research team member also recorded the time of the start and end of every lap and the location for the entire 20-minute walk while following the participant closely. The distance from the start of the trial to the 20th-minute mark was recorded after every trial using a distance measuring wheel (ML1212, Komelon, Waukesha, WI).

### 2.4 Data analysis

Spatiotemporal and EMG data from assessment visits were assessed for the last full lap for all participants. This was done to ensure that participants encountered a consistent number of different terrains and maximize their acclimation within the trial. Gait events (heel strikes and toe offs) were identified using accelerometer data from EMG sensors. Walking speed was calculated by dividing the length of the walking route by the time taken to complete the lap. The stride length was determined by dividing the length of the route by the number of strides completed in the lap. Cadence was calculated by dividing the lap time by the number of strides completed in the lap. EMG data were bandpass-filtered between 15 and 380 Hz, rectified, and low-pass-filtered at 7 Hz to create a linear envelope (Lerner et al., 2016). The filtered EMG data were divided into gait cycles, which were subsequently normalized to a scale of 0 - 100%. We then normalized the filtered EMG using the peak EMG value from the no-exo condition. Integrated EMG (iEMG) was calculated as the area under the mean (average of all gait cycles) EMG curve for the stance phase of the gait cycle for the soleus muscle and the swing phase of the gait cycle for the tibialis anterior muscle to represent muscle work (Bouisset and Goubel, 1973). We calculated the co-contraction between the soleus and tibialis anterior muscles using the co-contraction index (CCI) approach outlined by Rudolph et al. (2000), which takes into consideration the temporal and magnitude components of an EMG signal (Knarr et al., 2012), as represented in Equation 3:

$$CCI=\sum_{i=1}^{100}\frac{LEMG\left(i\right)}{MEMG\left(i\right)}\left(LEMG\;\left(i\right)+MEMG\;\left(i\right)\right),$$ (3)

where *i* is the number of timepoints within the stance phase of the gait cycle, *LEMG* is the normalized magnitude of the less active muscle at time point *i*, and *MEMG* is the normalized magnitude of the more active muscle at time point *i*. iEMG and CCI were calculated for the more affected limb.

### 2.5 Statistical analysis

To test our *a priori* hypotheses that walking with exoskeleton assistance and real-world gait training would improve spatiotemporal and muscle activity outcomes, we compared walking conditions within each assessment visit *(pre exo* vs*. pre no-exo; post exo* vs*. post no-exo)* and across assessment visits *(pre* vs*. post exo; pre* vs*. post no-exo)*. We also compared acclimated exoskeleton-assisted walking *(post exo)* to pre-intervention baseline performance *(pre no-exo)*. We examined all datasets for normality by performing the Shapiro–Wilk goodness-of-fit test. Paired two-tailed t-tests were used to evaluate any changes for normally distributed comparisons, while the Wilcoxon signed-rank test was used for non-normally distributed data. In *post hoc* exploratory analyses, we used linear regression to assess potential associations between stride length and walking speed, cadence and walking speed, and co-contraction at the ankle and walking speed. Two participants (P7 and P8) were excluded from EMG analyses for comparisons involving *post exo* because their sensor connection was lost during data collection. See Table 2 for details on the number of participants included in each comparison Statistical significance ( ∝ ) was set at *p* < 0.05 for all tests. We calculated the effect size (ES) using Cohen’s d for paired t-tests and r values for Wilcoxon signed-rank tests; an effect size less than 0.2 was considered small, between 0.5 and 0.8 was considered medium, and greater than 0.8 was considered large (Cohen, 1988). We also aimed to determine whether any observed significant differences met the threshold for the minimum clinically important differences (MCIDs), as defined by Oeffinger et al. (2008). To meet the MCID criteria for significant differences with large effect sizes, there had to be a 9.1%, 5.8%, and 8.1% improvement in walking speed, stride length, and cadence, respectively, relative to the no-intervention condition.

**TABLE 2:** Summary of statistical analysis with the number of samples (n) ^a^

Within visits	Across visits	N
Pre-no-exo vs. pre-exo	Pre-no-exo vs. post-no-exo	8
Post-no-exo vs. post-exo	Pre-exo vs. post-exo	6

^a^ For all outcome measures except walking speed. Walking speed had n = 8 for all comparisons since it did not depend on EMG sensor data.

## 3 Results

**FIGURE 2:**
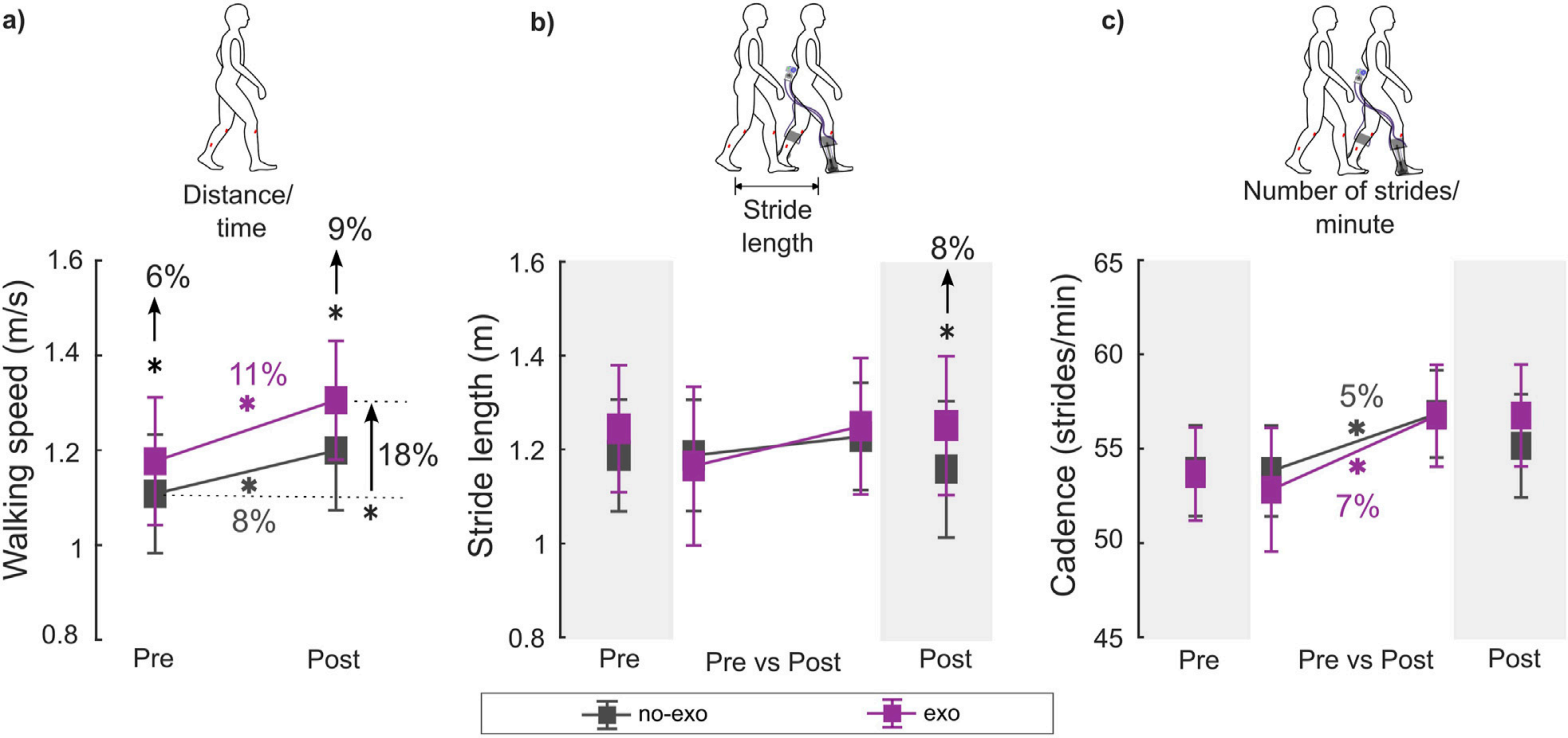
**(A)** Walking speed, **(B)** stride length, and **(C)** cadence during the pre- and post-assessments for each condition. Stride length and cadence were unavailable for two participants (P7 and P8) for the post-assessment exo condition due to wireless data transmission failure. Therefore, within-visit comparisons [*pre exo* vs*. pre no-exo (n = 8); post exo* vs*. post no-exo (n = 6)*] are shown on the darker gray background, while the across-visit comparisons [*pre* vs*. post exo (n = 6); pre* vs*. post no-exo (n = 8)*] are shown on the lighter gray background (see details in Table 2). * indicates a significant difference between conditions and/or visits. Error bars represents the standard error of the mean.

**FIGURE 3:**
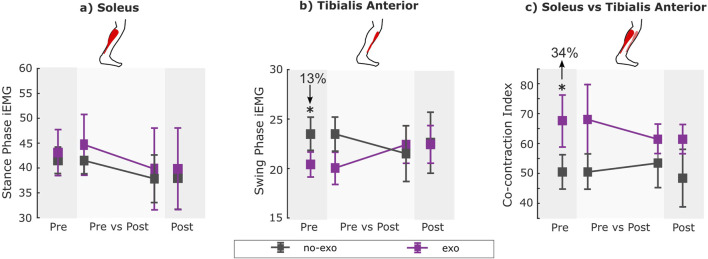
**(A)** Stance phase iEMG for soleus, **(B)** swing phase iEMG for tibialis anterior, and **(C)** co-contraction index (CCI) for all conditions across visits. EMG data were unavailable for two participants (P7 and P8) for the post-assessment exo condition due to wireless data transmission failure. Therefore, within-visit comparisons [*pre exo* vs*. pre no-exo (n = 8); post exo* vs*. post no-exo (n = 6)*] are shown on the darker gray background, while the across-visit comparisons [*pre* vs*. post exo (n = 6); pre* vs*. post no-exo (n = 8)*] are shown on the lighter gray background (see details in Table 2). * indicates a significant difference between conditions and/or visits. Error bars represent the standard error of the mean.

All participants walked and navigated the outdoor route with and without the device, without any adverse events. All participants walked faster with the ankle exoskeleton relative to the no-exo condition within both visits. Participants also walked faster in both assisted and unassisted conditions after training vs pre-training (Figure 2A). Participants also had similar muscle activity while walking with and without the exoskeleton except on the pre-assessment visit (Figure 3; Supplementary Figures S1, S2).

### 3.1 Spatiotemporal outcomes

In the pre-assessment, walking with the ankle exoskeleton resulted in a 6% increase in walking speed relative to the no-exo condition (*p* = 0.049; Supplementary Table S1).

Following training (i.e., post-assessment), exoskeleton-assisted walking speed increased by 11% and cadence by 7% compared to the pre-assessment, exceeding the MCID threshold of 9.1% for walking speed (Oeffinger et al., 2008) (*p* = 0.003 and *p* = 0.006; Supplementary Table S2). Unassisted walking speed also increased by 8%, with a 5% increase in cadence compared to the pre-assessment (*p* = 0.009 and *p* = 0.012; Supplementary Table S3).

In the post-assessment, walking with the ankle exoskeleton increased walking speed by 9% and stride length by 8% relative to the no-exo condition (*p* < 0.001 and *p* < 0.001; Supplementary Table S4). Walking speed improvement met the MCID threshold of 9.1%, while stride length exceeded the 5.8% threshold (Lerner et al., 2016).

Comparing the post exo condition (i.e., acclimated exoskeleton-assisted walking) with the pre no-exo condition (i.e., baseline), participants walked 18% faster with a 10% increase in stride length and a 9% increase in cadence (*p* < 0.001, *p* < 0.001, and *p* = 0.04; Supplementary Table S5). These improvements in walking speed, stride length, and cadence exceeded the MCID for each outcome (Oeffinger et al., 2008).

### 3.2 Muscle activity

In the pre-assessment, swing phase TA iEMG decreased by 13%, while the co-contraction index increased by 34% when walking with the exoskeleton relative to without assistance (*p* = 0.02 and *p* = 0.02; Supplementary Table S1).

Following training (i.e., post-assessment), stance-phase soleus iEMG, swing-phase TA iEMG, and co-contraction index during both assisted and unassisted walking were similar compared to those during pre-assessment (Supplementary Tables S2, S3).

In the post-assessment, walking with the exoskeleton resulted in similar stance-phase soleus iEMG, swing-phase TA iEMG, and co-contraction index compared to walking without assistance (Supplementary Table S4).

Comparing the post-exo condition (i.e., acclimated exoskeleton-assisted walking) with the pre no-exo condition (i.e., baseline), walking with the exoskeleton resulted in similar stance-phase soleus iEMG, swing-phase TA iEMG, and co-contraction index compared to walking without assistance (Supplementary Table S5).

### 3.3 Associations between spatiotemporal and muscle activity outcomes

**FIGURE 4:**
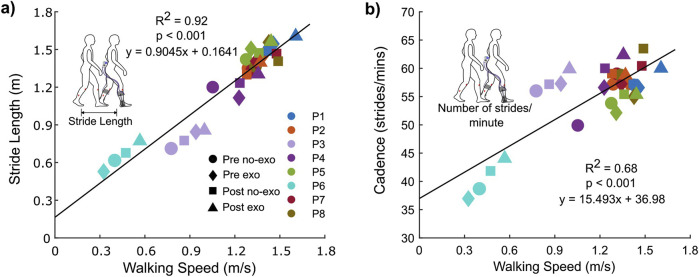
Association between walking speed and **(A)** stride length and **(B)** cadence for both conditions across visits.

Walking speed had a significant association with stride length and cadence (Figure 4). There was a strong and significant association between the walking speed and stride length (*R* ^2^ = 0.92 and *p* < 0.001; Figure 4A); participants took longer strides at faster speeds. Cadence explained 68% of the variance in walking speed (*R* ^2^ = 0.68 and *p* < 0.001; Figure 4B).

## 4 Discussion

The benefits of exoskeleton assistance in the laboratory or controlled overground settings for those with CP and other impaired populations are well-documented (Buesing et al., 2015; Orekhov et al., 2020; Fang et al., 2022; Srivastava et al., 2015; Lerner et al., 2017; Conner B. C. et al., 2021; Awad et al., 2017). We previously demonstrated that ankle exoskeleton assistance can improve the walking economy and speed of individuals with CP on mixed real-world terrain (Tagoe et al., 2024). However, we are not aware of any study investigating how exoskeleton use in real-world settings affects spatiotemporal and neuromuscular outcomes for any impaired population. Knowledge of the mechanisms behind the improved performance with exoskeleton assistance in real-world settings can guide future interventions and treatment strategies in CP. Our hypotheses were partially confirmed; walking speed and stride length increased after short-term, low-frequency exoskeleton-assisted training and when walking with assistance (vs without); co-contraction between the soleus and the tibialis anterior muscles, however, was not significantly different after training.

In this study, individuals with CP safely walked and navigated in the ankle exoskeleton in real-world settings without any adverse events, demonstrating a 6%–18% increase in walking speed with exoskeleton assistance. This improvement was more than the 5%–9% increase observed in a real-world exoskeleton-assisted study with healthy individuals (Slade et al., 2022). In that study, the exoskeleton used a human-in-the-loop optimization process that required approximately an hour of specific user walking data prior to walking trials to prescribe optimal assistance. In contrast, our exoskeleton’s proportional joint-moment control strategy did not require user data or manual fine-tuning before walking trials while still acknowledging differences in study protocol and participant populations.

Increasing walking speed is crucial for increasing social activity and participation levels for individuals with CP. It remains an important clinical goal of functional gait training for effective CP rehabilitation (Todd et al., 1989; Hoffman et al., 2018; Booth et al., 2018; Moreau et al., 2016). In this study, robot-assisted gait training led to significant improvements in walking speed in real-world settings, with an 11% increase during assisted walking and an 8% during unassisted walking. This could suggest that while familiarization with the terrain likely contributed to these improvements, gait training with ankle assistance also promotes faster ambulatory speeds. It also appears that these improvements can be reinforced over time, even after discontinuing use of the device. Future studies should include a control group that completes only unassisted walking to better isolate the effect of exoskeleton assistance from that of terrain familiarization.

Increasing stride length and cadence is a strategy for increasing the gait speed in both individuals with CP and typically developing individuals (Abel and Damiano, 1996). This study supports this concept as there was a significant association between walking speed and both stride length and cadence (Figures 4A, B). Historically, individuals with CP have tended to increase their walking speed by increasing cadence rather than stride length (vs typically developing individuals) (Abel and Damiano, 1996; Davids et al., 2019; Todd et al., 1989). The results from this study showed that increased stride length explained 92% of the variance in walking speed, as opposed to 68% for cadence. While not claiming causation, these associations suggest that ankle exoskeleton assistance may facilitate a more natural strategy for increasing speed by emphasizing longer steps rather than more frequent steps.

Short-term, low-frequency, real-world gait training resulted in increased walking speed, stride length, and cadence that exceeded MCID with medium-to-large effect sizes necessary for clinical translation (Oeffinger et al., 2008). MCID is a standard way of measuring and assessing the clinical relevance and efficacy of an intervention (Copay et al., 2007). It goes beyond statistical significance to reflect the smallest improvement that a patient can perceive as beneficial or meaningful (Oeffinger et al., 2008; Jaeschke et al., 1989; Wright et al., 2012; Storm et al., 2020). Thus, the results of this study indicate that utilizing this ankle exoskeleton assistance in real-world settings can result in meaningful improvements to participants’ functional ability, further reinforcing the potential of this assistive tool as a transformative rehabilitation device.

Somewhat surprisingly, acclimated exoskeleton-assisted walking resulted in no significant changes in muscle activity outcomes after training. Moreover, exoskeleton assistance led to higher co-contraction during the pre-assessment visit. This initial increase in co-contraction may suggest that participants were unacclimatized to the device, potentially leading to a greater need for balance or ankle stability as they attempted to adapt (Di Nardo et al., 2015). The return to similar muscle activity after training, even at higher speeds, might reflect some form of acclimation. However, the lack of significant improvement, following training, could indicate that participants had not fully adapted to the device. Further research is needed to explore whether a longer acclimation period or higher frequency training could better enhance the benefits of exoskeleton assistance on muscle activity in real-world settings.

Our study had several limitations. First, we had a relatively small sample size (n = 8), so it is important not to overgeneralize our findings. Second, due to the absence of a control group in this study, we cannot fully isolate the benefits of exoskeleton assistance from those of terrain familiarization. Future studies should include a control group for comparison with the intervention group. Furthermore, this was a feasibility study that evaluated the benefits of the exoskeleton assistance after only four low-frequency exoskeleton-assisted walking sessions. Hence, more and higher-frequency training sessions could potentially result in greater improvements. Future work should aim to increase the number and frequency of sessions to explore the full extent of exoskeleton assistance. Finally, this study assessed overall performance across the entire path without analyzing the specific performance variations across each terrain type. Future studies should examine these variations to determine how exoskeleton assistance performs on each terrain in the real world.

## 5 Conclusion

In summary, this feasibility study demonstrates that short-term, low-frequency gait training with an untethered, lightweight ankle exoskeleton can safely and effectively improve spatiotemporal outcomes in individuals with CP and diverse lower-limb impairments when performed on real-world terrain. Training led to clinically relevant improvements in walking speed, stride length, and cadence but did not lead to changes in the co-contraction of the soleus and tibialis anterior muscles. This study supports further research to evaluate the performance of robotic training interventions over real-world terrain for individuals with CP.

## Data Availability

The raw data supporting the conclusions of this article will be made available by the authors, without undue reservation.
