# Supplementary material for: Exoskeleton gait training on real-world terrain improves spatiotemporal performance in cerebral palsy
Source: Front Bioeng Biotechnol. 2024 Dec 17;12:1503050. doi: 10.3389/fbioe.2024.1503050 (PMC11685018; doi:10.3389/fbioe.2024.1503050)
Supplement: Supplementary file 1 [file DataSheet1.docx]

Supplementary Material

# Supplementary Figures and Tables

## Supplementary Figures


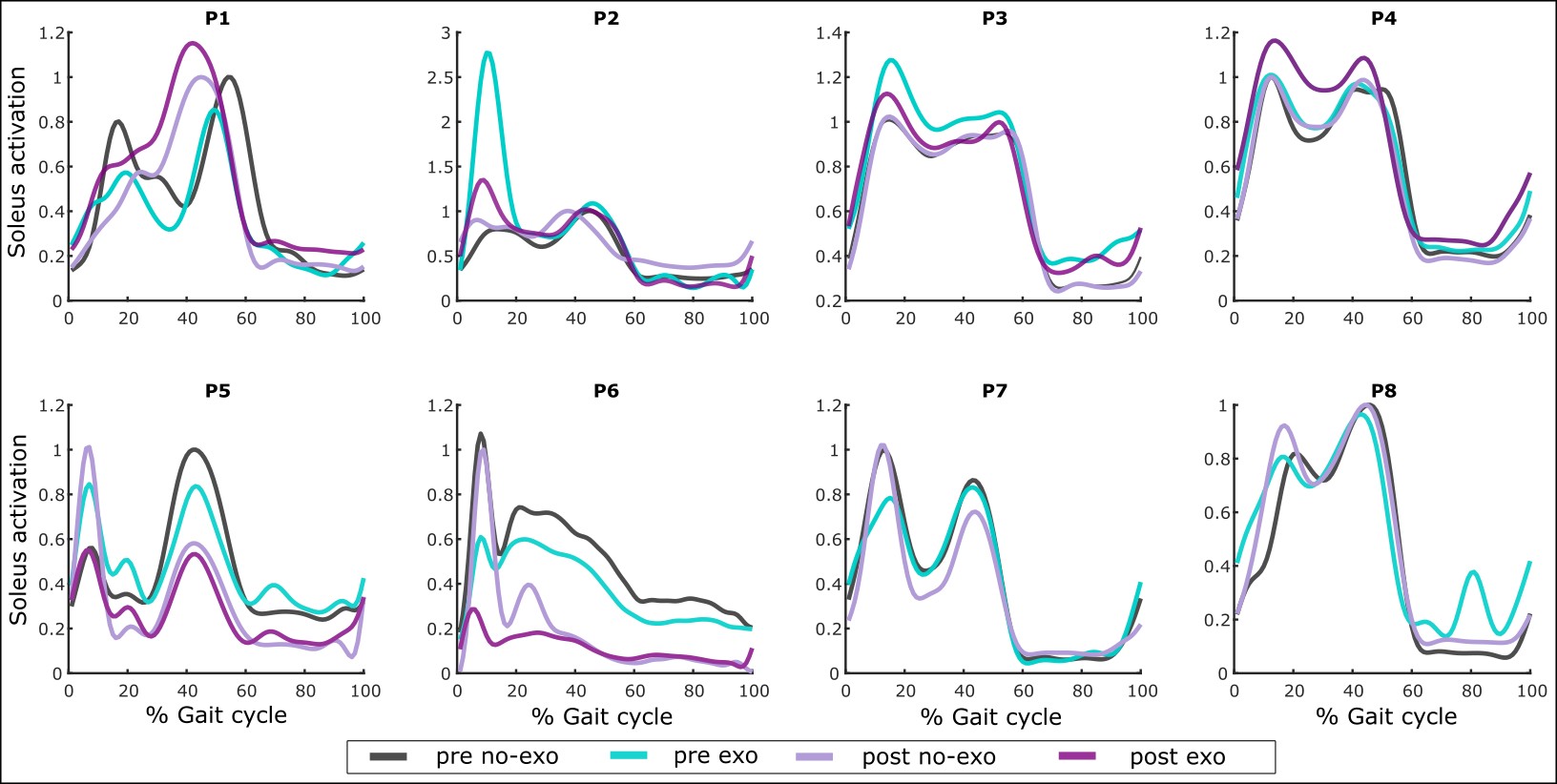


**Supplementary Figure 1.** Mean (average of all gait cycles) soleus muscle activation for each participant for all conditions across visits. EMG data were unavailable for two participants (P7 and P8) for the post assessment exo condition due to wireless data transmission failure.


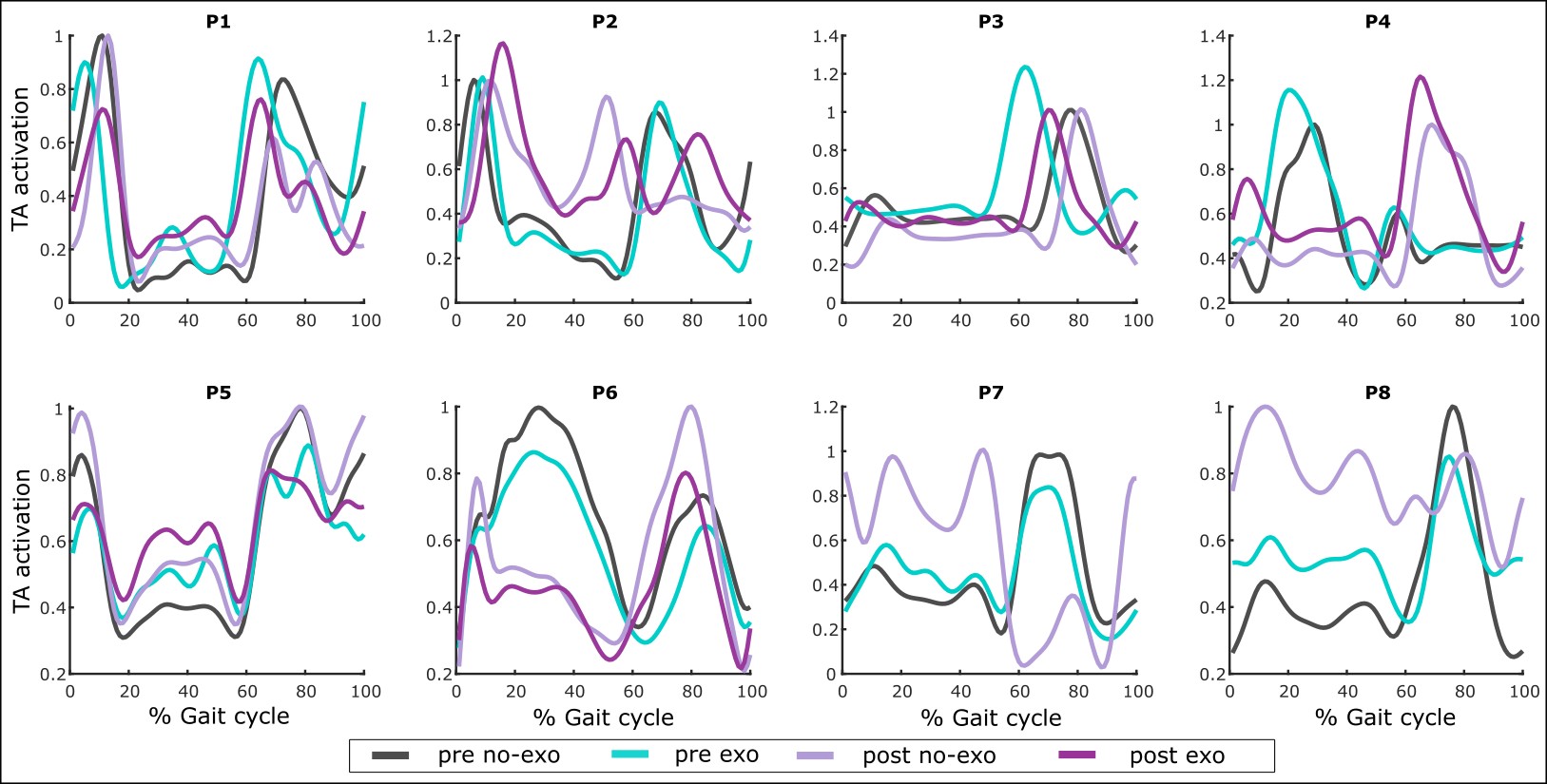


**Supplementary Figure 2.** Mean (average of all gait cycles) tibialis anterior muscle activation for each participant for all conditions across visits. EMG data were unavailable for two participants (P7 and P8) for the post assessment exo condition due to wireless data transmission failure.

## Supplementary Tables

**Supplementary Table 2.** Results of pairwise comparison of all outcome measures between conditions during pre-assessment. Bold blue values represent statistical significance.

|  | Outcome measures | Pre no-exo  Mean $\boldsymbol{\pm}$ SEM | Pre exo  Mean $\boldsymbol{\pm}$ SEM | Difference  Mean $\boldsymbol{\pm}$ SEM | p-value | Effect size |
| --- | --- | --- | --- | --- | --- | --- |
| Spatiotemporal Outcomes | Walking speed  (m/s) | 1.11 $\pm$ 0.12 | 1.18 $\pm$ 0.13 | 0.07 $\pm$ 0.03 | **0.049** | 0.8 |
|  | Stride Length  (m) | 1.19 $\pm$ 0.12 | 1.24 $\pm$ 0.13 | 0.06 $\pm$ 0.03 | 0.149 | 0.5 |
|  | Cadence  (strides/mins) | 53.82 $\pm$ 2.40 | 53.64 $\pm$ 2.46 | 0.17 $\pm$ 1.12 | 0.881 | 0.05 |
| Muscle Activity | Stance Phase iEMG (Soleus) | 41.50 $\pm$ 2.64 | 43.08$\pm$4.61 | 1.58$\pm$2.78 | 0.59 | 0.20 |
|  | Swing Phase iEMG (TA) | 23.52$\pm$1.72 | 20.45$\pm$1.27 | 3.07 $\pm$1.02 | **0.02** | 1.06 |
|  | Co-contraction Index | 50.53 $\pm$ 5.76 | 67.68 $\pm$ 8.72 | 17.15$\pm$5.74 | **0.02** | 1.06 |

**Supplementary Table 2.** Results of pairwise comparison of all outcome measures for the exoskeleton-assisted walking condition during pre-assessment vs post-assessment. Bold blue values represent statistical significance.

|  | Outcome measures | Pre exo  Mean $\boldsymbol{\pm}$ SEM | Post exo  Mean $\boldsymbol{\pm}$ SEM | Difference  Mean $\boldsymbol{\pm}$ SEM | p-value | Effect size |
| --- | --- | --- | --- | --- | --- | --- |
| Spatiotemporal Outcomes | Walking speed  (m/s) | 1.18 $\pm$ 0.13 | 1.31 $\boldsymbol{\pm}$ 0.13 | 0.13 $\boldsymbol{\pm}$ 0.03 | **0.003** | 1.6 |
|  | Stride Length  (m) | 1.16 $\pm$ 0.17 | 1.25 $\pm$ 0.15 | 0.08 $\pm$ 0.05 | 0.122 | 0.8 |
|  | Cadence  (strides/mins) | 52.82 $\pm$ 3.27 | 56.74 $\pm$ 2.70 | 3.92 $\pm$ 0.86 | **0.006** | 1.9 |
| Muscle Activity | Stance Phase iEMG (Soleus) | 44.70$\pm$6.05 | 39.81$\pm$8.22 | 4.89$\pm$5.14 | 0.39 | 0.39 |
|  | Swing Phase iEMG (TA) | 20.01$\pm$1.66 | 22.41$\pm$1.90 | 2.36$\pm$2.06 | 0.30 | 0.47 |
|  | Co-contraction Index | 68.03$\pm$11.60 | 61.43$\pm$4.88 | 6.60$\pm$9.04 | 0.50 | 0.30 |

**Supplementary Table 3.** Results of pairwise comparison of all outcome measures for the unassisted walking condition during pre-assessment vs post-assessment. Bold blue values represent statistical significance.

|  | Outcome measures | Pre no-exo  Mean $\boldsymbol{\pm}$ SEM | Post no-exo  Mean $\boldsymbol{\pm}$ SEM | Difference  Mean $\boldsymbol{\pm}$ SEM | p-value | Effect size |
| --- | --- | --- | --- | --- | --- | --- |
| Spatiotemporal Outcomes | Walking speed  (m/s) | 1.11 $\pm$ 0.12 | 1.20 $\boldsymbol{\pm}$ 0.13 | 0.09 $\boldsymbol{\pm}$ 0.03 | **0.009** | 1.3 |
|  | Stride Length  (m) | 1.19 $\pm$ 0.12 | 1.23 $\pm$ 0.11 | 0.04 $\pm$ 0.02 | 0.076 | 0.7 |
|  | Cadence  (strides/mins) | 53.82 $\pm$ 2.40 | 56.84 $\pm$ 2.31 | 3.02 $\pm$ 1.11 | **0.012** | 0.9 |
| Muscle Activity | Stance Phase iEMG (Soleus) | 41.50$\pm$2.64 | 37.86$\pm$4.77 | 3.64$\pm$2.77 | 0.23 | 0.47 |
|  | Swing Phase iEMG (TA) | 23.52$\pm$1.72 | 21.54$\pm$2.83 | 1.98$\pm$2.55 | 0.46 | 0.27 |
|  | Co-contraction Index | 50.53 $\pm$ 5.76 | 53.51 $\pm$ 8.19 | 2.98$\pm$11.11 | 0.80 | 0.10 |

**Supplementary Table 4.** Results of pairwise comparison of all outcome measures between conditions during post-assessment. Bold blue values represent statistical significance

|  | Outcome measures | Post no-exo  Mean $\boldsymbol{\pm}$ SEM | Post exo  Mean $\boldsymbol{\pm}$ SEM | Difference  Mean $\boldsymbol{\pm}$ SEM | p-value | Effect size |
| --- | --- | --- | --- | --- | --- | --- |
| Spatiotemporal Outcomes | Walking speed  (m/s) | 1.20 $\boldsymbol{\pm}$ 0.13 | 1.31 $\boldsymbol{\pm}$ 0.13 | 0.11 $\boldsymbol{\pm}$ 0.01 | **<0.001** | 2.7 |
|  | Stride Length  (m) | 1.16 $\pm$ 0.14 | 1.25 $\pm$ 0.15 | 0.09 $\pm$ 0.01 | **<0.001** | 5.9 |
|  | Cadence  (strides/mins) | 55.13 $\pm$ 2.74 | 56.74 $\pm$ 2.70 | 1.61 $\pm$ 0.56 | 0.116 | 0.6 |
| Muscle Activity | Stance Phase iEMG (Soleus) | 37.93$\pm$6.35 | 39.81$\pm$8.22 | 1.88$\pm$2.86 | 0.54 | 0.27 |
|  | Swing Phase iEMG (TA) | 22.59$\pm$3.10 | 22.41$\pm$1.90 | 0.18$\pm$2.01 | 0.93 | 0.04 |
|  | Co-contraction Index | 48.41 $\pm$ 9.60 | 61.43 $\pm$ 4.88 | 13.02$\pm$10.56 | 0.27 | 0.50 |

**Supplementary Table 5.** Results of pairwise comparison of all outcome measures between the unassisted walking condition during pre-assessment vs exoskeleton assisted walking condition during post-assessment. Bold blue values represent statistical significance.

|  | Outcome measures | Pre no-exo  Mean $\boldsymbol{\pm}$ SEM | Post exo  Mean $\boldsymbol{\pm}$ SEM | Difference  Mean $\boldsymbol{\pm}$ SEM | p-value | Effect size |
| --- | --- | --- | --- | --- | --- | --- |
| Spatiotemporal Outcomes | Walking speed  (m/s) | 1.11 $\pm$ 0.12 | 1.31 $\boldsymbol{\pm}$ 0.13 | 0.20 $\boldsymbol{\pm}$ 0.02 | **<0.001** | 3.1 |
|  | Stride Length  (m) | 1.13 $\pm$ 0.15 | 1.25 $\pm$ 0.15 | 0.12 $\pm$ 0.02 | **<0.001** | 2.9 |
|  | Cadence  (strides/mins) | 52.11 $\pm$ 2.90 | 56.74 $\pm$ 2.70 | 4.63 $\pm$ 1.68 | **0.040** | 1.1 |
| Muscle Activity | Stance Phase iEMG (Soleus) | 42.43$\pm$3.47 | 39.81$\pm$8.22 | 2.62$\pm$6.00 | 0.68 | 0.18 |
|  | Swing Phase iEMG (TA) | 23.22$\pm$2.30 | 22.41$\pm$1.90 | 0.81$\pm$2.46 | 0.76 | 0.13 |
|  | Co-contraction Index | 54.57$\pm$7.00 | 61.43$\pm$4.88 | 6.86$\pm$5.92 | 0.30 | 0.47 |
